# Authors’ reply

**Published:** 2010

**Authors:** Ramesh Murthy, Siddharth Kesarwani

**Affiliations:** 1 Pediatric Ophthalmology and Strabismus, Oculoplasty, Hyderabad, India; 2 Orbital diseases and Ocular Oncology, LV Prasad Eye Institute, Hyderabad, India

Dear Editor,

We are grateful for the comments and issues raised in the letter by Pandey *et al*.[1] We performed botulinum toxin injections to both lateral recti (not medial recti as mentioned in the comments) of this young woman with head injury, with subsequent recovery of fusion.[2] We agree that central motor fusion deficiency (CFD) is a diagnosis of exclusion and we have excluded all possible causes before labeling the entity as CFD. Internuclear ophthalmoplegia (INO) presents with limited adduction and abducting nystagmus, which was not present in our patient; convergence / accommodative paresis presents with poor near vision, which was preserved in our patient; saccadic intrusions / oscillations were unlikely as there were no involuntary movements noticed on examination; pre-existing decompensated phoria was again unlikely as there was no history indicative of this, although decompensation occurs if fusion is disrupted, due to decreased vision in one eye; and finally a monofixation syndrome was unlikely considering 20/20 vision in each eye. Adduction was full excluding a diagnosis of bilateral INO.

Central motor fusion deficiency is diagnosed only when all other causes are reasonably excluded. Small hypertropia, bilateral extorsion along with vertical bobbing of a nonfusible image on attempted fusion may not be seen in all cases.[3] In fact there are more cases of CFD reported without this finding than with this finding. Magnetic resonance imaging was performed, but did not reveal any lesion in the region of the median longitudinal fasciculi. Restoration of fusion in CFD has been reported following squint surgery.[4] We agree that prisms and orthoptic exercises cannot be expected to restore fusion. The difference with botulinum toxin is, the alignment is maintained for a reasonable period of time, which is why the treatment helps.

Our patient did not have disruption of uniocular vision at any time. Newer imaging modalities are being tried to find a functional deficit in the absence of anatomical lesions. Our diagnosis is a diagnosis of exclusion after ruling out the common presentations of other differentials. The fact remains that if fusable images are presented to both eyes for a prolonged period of time by restoring motor alignment, the central nervous system might regain the ability to fuse the images.
